# Seeding strategies for new product launch: The role of negative word-of-mouth

**DOI:** 10.1371/journal.pone.0206736

**Published:** 2018-11-05

**Authors:** Fang Cui, Hai-hua Hu, Wen-tian Cui, Ying Xie

**Affiliations:** 1 School of Management, Xi’an Jiaotong University, Xi’an, China; 2 School of Management, Xi’an University of Architecture and Technology, Xi’an, China; 3 School of Economic & Management, Northwest University, Xi’an, China; Centre National de la Recherche Scientifique, FRANCE

## Abstract

When launching a new product, firms often give away free samples to seed the market. This paper aims to identify the optimal seeding targets, such as early adopters, social hubs, or randomly chosen consumers while considering the presence of negative word-of-mouth (WOM). Using agent-based modeling, it was found that seeding early adopters can generate the highest profit and the largest market penetration, followed by the social hubs and random consumers. Moreover, the results show that seeding early adopters can be more beneficial for a low-quality product, wherein adopters are more likely to spread negative WOM. These findings challenge a widely accepted notion in the related research that social hubs are often the most promising targets for seeding programs.

## Introduction

Seeding is the giving away of a new product to a part of consumers (the “seeds”) before launching and is a common approach to promote the diffusion process in various industries, such as software, electronic and automakers. For instance, Microsoft distributed Windows 95 to *ca*. 5% of potential consumers of USA in 1995 [1]. Sony seeded the market with PlayStation products worth 1 million US dollars in 2009 [2]. Ford selected 100 Ford Fiesta’s target consumers from the bloggers, and let them make car reviews in the forum [3]. Against this backdrop, seeding strategies have been of great academic interest, especially over the last decade [4–7].

In this paper, an attempt has been made to identify the best targets for the seeding programs. In this regard, several options are in common use. For example, marketers often ignore market segment differences and select seeds randomly, as seen in the case of Microsoft’s Windows 95 distribution [1]. Alternatively, marketers place focus on influential people with deep social connections (referred to as social hubs) and utilize their wide-ranging influence to ignite the market, as seen in the case of Ford, who used prominent bloggers [3]. Another option is the early adopters, who have high intention to purchase the firm’s product, typically loyal customers, as Sony did for the launch of PlayStation[2]. Accordingly, the current study primarily focuses on three options, namely, the social hubs, early adopters, and random consumers.

In fact, some studies have done the same investigation, such as Nejad et al. and Hu et al. [7,8]. However, these studies only emphasize the effect of positive word-of-mouth (WOM) associated with the seeding programs. The current study further considers the presence of negative WOM in the product-diffusion processes. Previous evidence shows that negative WOM is not only more persuasive for a consumer’s purchasing decisions than positive WOM, but also propagates faster [9–12]. This implies that negative WOM can remarkably change and slow down the product-diffusion process. Some researchers even attribute abundant failures of commercialized innovations to negative WOM [13–15]. Therefore, ignoring negative WOM may lead to unexpected results from the seeding programs.

More importantly, the presence of negative WOM may challenge the widely accepted idea that social hubs are the most promising targets before any other type of consumers. It is common understanding that the strength of social hubs comes from the fact that they can influence more users and therefore, can drive marketing buzz more effectively. However, with the presence of negative WOM, such strength can also become a weakness, which means that they also facilitate the spread of negative WOM. It could be worse when seeding social hubs, because it possibly assists negative WOM in penetrating the market at the early stage of product diffusion process. Along this logic, seeding early adopters seems to become more promising, as they exhibit a significantly stronger intention to spread positive WOM [16]. The results show that the best option of seeding targets varies between scenarios of with and without negative WOM.

In this work, agent-based modeling and simulation (ABMS) have been used to address the research objective. Admittedly, building experiments based on real data is more appropriate and credible. However, conducting seeding programs in the real market is too complex and expensive. Moreover, negative WOM often leaves few clues in the sales data. By contrast, ABMS is not limited to observed data, and provides the ability to simulate the market by capturing consumers as agents, who interact through a social network. Based upon this approach, the market response is approximated under various conditions, which are associated with the product and the consumers, and then, the performance of different seeding targets is compared. The results showed that, in the presence of negative WOM, seeding early adopters can generate the highest profit and the largest market penetration on average, followed by social hubs and random consumers. Based on the study of Nejad [8], the effect of homophily on seeding performance is considered in the presence of negative WOM. It is found that, due to the influence of negative WOM, the impact of homophily on seeding performance changes.

The remainder of this paper is organized as follows. The literature review is provided in Section 2. In Section 3, the ABMS model is constructed and the rule of adoptions is described. Section 4 provides the results. Finally, the theatrical and managerial implications are discussed and the deficiency and the extent of potential of this paper are presented.

## Literature review

### Negative WOM in diffusion

When the product does not meet the consumers’ expectation, the consumers may feel dissatisfied. According to the surveys of Sweden and the US, extremely dissatisfied consumers share the negative attitude with more than ten friends [17]. In the fashion industry, the majority of dissatisfied consumers spread negative WOM to five peers on average [12]. Charlett and Garland (1995) showed the spreading evidence of negative WOM [18]. The consumers’ expectation will be affected by retail price, description of the advertisement [19], WOM from peers, quality of the new product and consumers’ propensity to adopt [16]. If the consumers’ attitude is negative, the consumer may take one or more of three actions: reject the product to purchase some other, complain to the firm, and spread negative WOM to peers [11,12,20,21]. The first two actions will not influence the other potential consumers in the social network, though the last one does.

Additionally, the recipients of WOM pay more attention to negative information [9], which is supposed to be more informative [10–12]. The impact of negative information can offset of the effect of positive WOM. Along with the popularization of Internet, the negative WOM can spread faster than in the traditional social network [22]. Therefore, the impact of negative WOM is non-linear, which makes it possible that even a small percentage of dissatisfied adopters could markedly decrease firms’ profits.

The impacts of mean and standard deviation of online ratings on pricing, demand, and profit have been well studied in prior literature. The average rating indicates the quality of a product and has a positive impact on the demand and profit [23]. Previous studies about the impact of negative WOM on diffusion process report that the percentage of dissatisfied adopters directly decreases the NPV (net present value) ratio and indirectly increases the rejections [24]. There is no study focusing on the effect of standard deviation of the probability of consumers spreading negative WOM on the diffusion process. Related researches about the online ratings have explored the informational role of standard deviation of product ratings, which is described as follows [25]. The results suggest that, a low standard deviation of rating indicates a mainstream product, which is designed to satisfy most of the consumers’ tastes. A high standard deviation of ratings corresponds to a niche product, which is the one that some consumers love, while the others hate. In this paper, the informational roles of mean and standard deviation of the probability of consumers spreading negative WOM are consistent with these results. According to some previous studies, the mean of the probability of consumers spreading negative WOM has a significant impact on a firm’s profit, whereas the standard deviation indicates the characteristics of a product. Based upon this, the current study incorporates both of these factors (probability and standard deviation) into the research.

### Seeding programs and selection of targets

When a new product, which is not known to the most consumers, enters the market, the firm will take some marketing strategies to introduce it to the potential consumers, and the most common practice is the advertising. Christophe and Van Den Bulte found that when the marketing efforts are controlled, contagion effects disappear [26]. This shows that, before 2000, advertising played a decisive role in the spread of new products. However, with the proliferation of advertising, consumers place less value on the information from marketing compared with that from their friends [4]. Therefore, firms conduct seeding programs, in which the new products are distributed free of charge to a selected part of potential consumers (named, ‘seeds’) before launching, and expect that the seeds can express positive WOM to their peers about the new product. Therefore, through seeding programs, a portion of the potential consumers of social network can receive information (WOM) about the new product from their friends at an early stage of its launch. Part of them adopt the new product and become new WOM senders to social network. This way, seeding can shorten the time of building a massive enough number of adopters, who can effectively contribute to the diffusion process. Libai and Muller (2013) have found that seeding can significantly enhance the diffusion process from the following two aspects. (1) Expansion: seeding can increase the number of unexpected consumers, who would not have purchased the product otherwise. (2) Acceleration: through seeding, consumers, who would have adopted anyway, will purchase the product ahead of time [6].

In order to enhance the performance of seeding, the managers and researchers tend to choose more appropriate targets. Generally, they select targets according to two characteristics of consumers, which are the position in the social network and the propensity to adopt. In the social network literature, who have large number of ties to other people, are called the influentials, opinion leaders, mavens or sometimes hubs, and they can accelerate the spread of new products [27–29]. Ignoring the negative WOM, the most commonly selected targets are social hubs, who have the most connections with others in the network, and are easily identified. Seeding social hubs has an advantage in terms of exposing the new product to a great number of potential consumers within a short period of time. Additionally, social hubs are inclined to connect the chasm between early adopters and others [30]. Seeding hubs can speed up the diffusion and expand the market penetration [31]. However, it is costly to maintain the social connections with peers, while the hubs may not make full use of their connections [4]. Therefore, another targeting strategy is proposed as seeding the consumers with high propensity to adopt, who are known as early adopters. It would help speed up the whole diffusion process and enhance the propagation of WOM at early stages [32]. Early adopters are more interested in the update of technology [33] and have the capacity to accept financial loss when the innovation fails [31,34].

Previous studies focusing on seeding programs have left out the consideration of negative WOM. In this study, the optimal seeding target is considered by comparing the performances of seeding early adopters, social hubs and randomly selected consumers. Furthermore, under different conditions of market and product, the performance of seeding different targets would change. Especially for the level of homophily, mean and standard deviation of probability of consumers spreading negative WOM, they could impact the optimal seeding target. Furthermore, the relationships of the factors and the seeding performance need to be re-examined with the presence of negative WOM.

## Construction of the ABMS model

### Overview of the ABMS

In this work, the non-linear effect of negative WOM on NPV and market penetration from micro (individual) to macro (aggregate) levels is studied. Constructing models based on empirical data is a reliable way, though it is difficult to capture the diffusion process of a large amount of negative WOM within a full social network in the real world. In order to solve this problem, ABMS (agent-based modeling and simulation) has been used to simulate the consumers as agents and the diffusion process over time, which offers the opportunity to obtain the propagation path of negative WOM. ABMS is used frequently in previous studies focusing on the diffusion process [6,8,13,24,35,36]. The rule of adoption of new product and the construction of the network of consumer agents are included in the ABMS model. All assumptions adopted in the process are supported by empirical results or the conclusions from previously published papers. Relying on it, a large number of simulation experiments with different combinations of parameters are performed, in which the properties of network and agents remain unchanged.

### Product adoption process

Following the basic logic of the Bass diffusion model, a stochastic cascade approach has been adopted to describe the new product’s diffusion process [15]. In accordance with the diffusion theory, the potential consumers make the adoption decision according to two groups of factors, namely the external and internal factors, which are represented by parameters *p* and *q*, respectively [37,38]. The external factors usually refer to the firms’ marketing activities, such as advertising, external communications, consumers’ innovativeness, and affordability [15,36,39,40]. At the individual level, the marketing activities are same for everyone, and therefore, the parameter *p* represents the propensity of each potential consumer to adopt the new product [36,39]. The parameter *q* captures the effects of WOM from every agent on others, who have direct connection with him/her.

**Fig 1:**
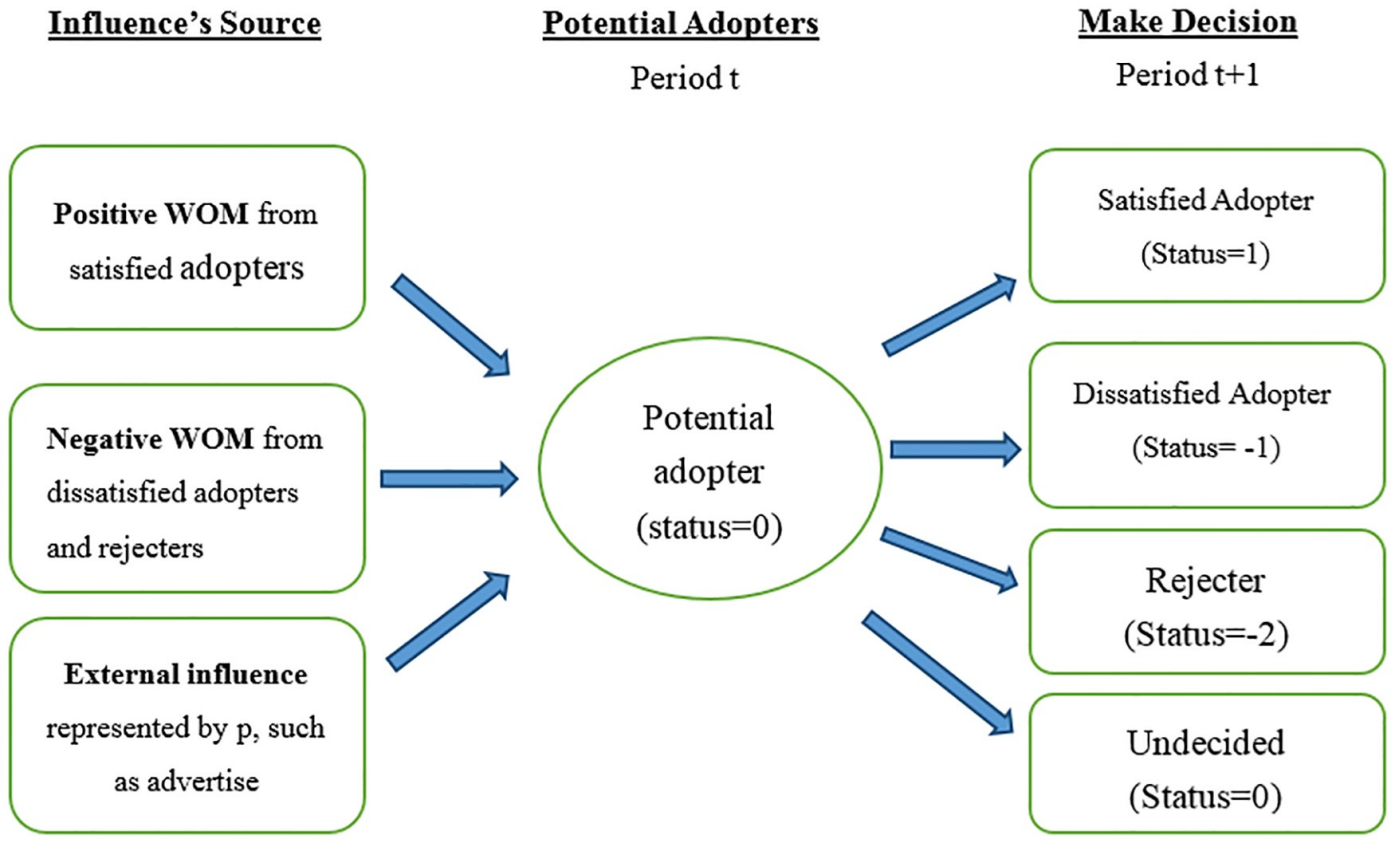
Decision making of potential consumers and the influences of WOM. Potential adopters are influenced by advertisement, and positive and negative WOMs. Each agent would be in one of the following states: the satisfied adopter, the dissatisfied adopter, the rejecter and the undecided consumer.

Each adopter has a particular probability of being dissatisfied with the product, after which, it spreads the negative WOM. As a result, once the potential consumer obtains the information about the new product, there may be four types of agents in the market namely the satisfied adopter, the dissatisfied adopter, the rejecter and the undecided consumer (Fig 1). Every agent stays in one of these four states. The satisfied adopter has adopted the new product and spreads positive WOM to his/her every connection because the adopter is pleased with the product. The dissatisfied adopter has adopted the new product and spreads negative WOM to all of its social connections because they are dissatisfied with the product. Confronting with the negative WOM from the dissatisfied adopters, some potential consumers directly become rejecters, who will never purchase the product. Rejecters also spread negative WOM to their connections. Some rejecters are influenced by the negative WOM from both dissatisfied adopters and the other rejecters connected with them [16,24]. The undecided consumers will not deliver WOM and still wait to obtain more information from marketing, such as advertising or the WOM from their connections. Moreover, potential adopter is undecided to adopt or reject the product and may change its state to any of the other three states based on WOM. In line with the previous literature, it is assumed that once the consumers have made their decisions and turned to one of the three states (satisfied adopter, dissatisfied adopter or rejecter), they will keep the states unchanged in future [24].

### Diffusion model

Every potential consumer will convert to one of the four states after receiving information about the new product with the probability of a change in state. Advertising intensity is same for every single individual, while the probability of change in state is decided by the propensity and the effects of WOM. First of all, the impacts of positive and negative WOMs on potential consumers need to be calculated. In particular, at each step t > 0, each potential consumer *i*, receives opinions from its neighbors in the network, such as positive WOM from satisfied adopters and negative WOM from dissatisfied adopters and rejecters. $C_{i}^{+1}\left(t\right)$, $C_{i}^{-1}\left(t\right)$ and $C_{i}^{-2}\left(t\right)$ represent the total number of satisfied adopters, total number of dissatisfied adopters and total number of rejecters connected with the agent *i* at period *t*, respectively. The influence of positive (negative) WOM on agent *i* (potential adopters) is determined based on the total number of satisfied adopters (dissatisfied adopters and rejecters), who connect with the agent *i* directly.

Thus, the agent *i*’s probability of being influenced by positive (or negative) WOM is determined using Eqs (1) and (2) [8,24].

$$p_{i}^{+}(t)=1-(1-p_{i})\prod_{j=1}^{C_{i}^{+1}(t)}\left(1-q_{j}\right)$$ (1)

$$p_{i}^{-}(t)=1-\prod_{j=1}^{C_{i}^{-1}(t)}\left(1-\text{m}q_{j}\right)\prod_{j=1}^{C_{i}^{-2}(t)}\left(1-mq_{j}\right)$$ (2)

where the parameter *m* represents the relative power of negative WOM to positive WOM. Since, the information recipients place more weight on negative WOM in making purchasing decision than the positive WOM [9,41,42], the parameter *m* is set at a reasonable level of 2, which is in line with the industrial practice [24,43].

Due to the influence of positive (or negative) WOM (represented by $p_{i}^{+}\left(t\right)$ and $p_{i}^{-}\left(t\right)$ respectively), the potential consumer *i* has a certain probability of changing opinion. More precisely, the probability of agent *i*’s adoption (or rejection) at period *t* is determined using Eqs (3)–(6) [24].

$$p_{i}^{\text{adp}}(t)=\left(1-p_{i}^{-}\left(t\right)\right)p_{i}^{+}\left(t\right)+\alpha_{i}p_{i}^{+}\left(t\right)p_{i}^{-}\left(t\right)$$ (3)

$$p_{i}^{\text{rej}}(t)=\left(1-p_{i}^{+}\left(t\right)\right)p_{i}^{-}\left(t\right)+(1-\alpha_{i})p_{i}^{+}\left(t\right)p_{i}^{-}\left(t\right)$$ (4)

$$p_{i}^{\text{undecided}}(t)=\left(1-p_{i}^{+}\left(t\right)\right)\left(1-p_{i}^{-}\left(t\right)\right)$$ (5)

and

$$\alpha_{i}=\frac{p_{i}^{+}\left(t\right)}{p_{i}^{+}\left(t\right)+p_{i}^{-}\left(t\right)}$$ (6)

Obviously, the sum of the three probabilities of adoption, rejection and undecided is 1. At every period *t*, the three probabilities for each potential agent are calculated, and after that, a uniform random number between 0 and 1 is generated to simulate the decision-making process.

The rejecters will spread negative WOM. The *d* parameter will decide if the adopted agent enters the satisfied or dissatisfied adopters class. The satisfied adopters would spread positive WOM to their direct connections, and the dissatisfied ones spread negative WOM. These WOMs impact the potential consumer’s adoption decision in the later periods. The *d* parameter is negatively correlated with the propensity to adopt (*p*_*i*_). Moldovan and Goldenberg invited 49 individuals to assess their attitude, purchasing intention, and WOM intentions with respect to four pre-launched products. The results show that the individuals, who expressed high purchasing intention, displayed a significantly stronger intention to spread positive WOM. On the contrary, individuals with low purchasing intention showed significantly stronger intention to spread negative WOM [16]. The results show: a) ‘high purchasing intention rated the innovation more favorably’, which means high propensity to adopt shows a high probability to spread positive WOM; b) ‘Subjects who rejected the innovation showed significantly stronger intention to spread negative WOM’, which means the consumers with low propensity to adopt have a low probability to spread positive WOM.

### Network model

The real-world social networks have several common properties. They have a short average path length, which means that, everyone in the network is located a few steps away from each other. Social networks have a high clustering coefficient. Clustering represents the “common friends are friends” tendency. These two features render a social network a small-world property [44].

The second property of many social networks is that people have unequal number of ties, which means that some people have more social contacts than others, thereby acting as “hubs” in the network [45,46]. In this study, the “free-scale” concept used by physicists has not been employed [46]. “Free-scale” depicts the phenomenon that many physical networks (such as, the World Wide Web) have a power-law degree distribution. However, such a distribution is clearly less likely to exist in social networks because the creation and maintenance of social contacts are costly. As proposed by Urry (2004), the degrees of people are almost normally distributed.

Another noteworthy property is the homophily, indicating that people tend to form social ties with alike people, which has an important influence on proliferation [47]. Centola has confirmed that social homophily constraints on tie formation generate emergent social topologies [48]. There are two types of homophily. One is the status homophily, which is based on similarities in the ascribed traits, such as race, age or gender and in the acquired traits, such as religion, education, or occupation. The other is the value homophily, which is based on value traits, such as attitudes or beliefs. According to McPherson et al. (2001) [49], value traits often prove to be derivative of status traits. Homophily plays an important role in the formation of collective actions and social movements [49]. The level of homophily affects the accuracy of the individuals’ estimates[50], which can help reach a consensus[51]. Moreover, about 50% contagion processes are attributed to homophily [52,53]. Homophily also plays an important role in retail management in addition to contagion process [54]. Consumers, who are connected closely, always make the same decision about whether to purchase a product or not, which is partly attributed to consumer homophily [55]. Consumers in the market with higher degree of homophily are more likely to have social connections with the people, who have similar propensity to adopt, which is exactly as the statement of homophily by Rogers [33]. As a first attempt, Nejad and Amini [8] explored the effect of homophily on the performance of three different seeding targets at a market level. The very explanation of homophily has been followed in the current work.

In order to reproduce the above structural properties as much as possible, a generative algorithm proposed by Hu et al. [7,56] is used. The first step is to generate *N* consumers with a propensity to adopt the new product (*q*), drawn from a normal distribution, which can be represented by: *q* ~ *N*(*μ*_*q*_, *σ*_*q*_). The second step is to build ties among the consumers. A tie will be added into the network at each time step. With the probability *h* ∈ [0,1], it connects two consumers, who have the most similar *q* and are not yet connected. With the probability *1-h*, it connects two individuals who are randomly selected from the population. In this case, self-loop and duplicating ties are forbidden. The pseudo-code of network formation is presented in Table 1.

**Table 1:** Pseudo-code for network formation.

Generate *N* agents
Assign the propensity of adoption *q* to each agent
For each tie Do
Select an agent *i* randomly from the population
Generate a random number *a*
If *a*<*h* Do
Select an agent *j* who has the most similar *q* to agent *i*
Else Do
Select an agent *j* randomly from the population
End If
% Avoid self-loop and overlapping
Add a tie between agents *i* and *j*
End For

Obviously, the parameter *h* implies the level of homophily and determines the structure of the network [56]. When *h = 0*, a uniform random network is yielded, exhibiting a small clustering coefficient and a short average path length. With the increase of *h*, the clustering coefficient and the average path length simultaneously increase. When *h = 1*, all ties are local and short, yielding a largely fragmented network. In this network model, the number of ties, which a consumer holds, always follows a Poisson distribution, and is irrespective of the value of *h*.

**Fig 2:**
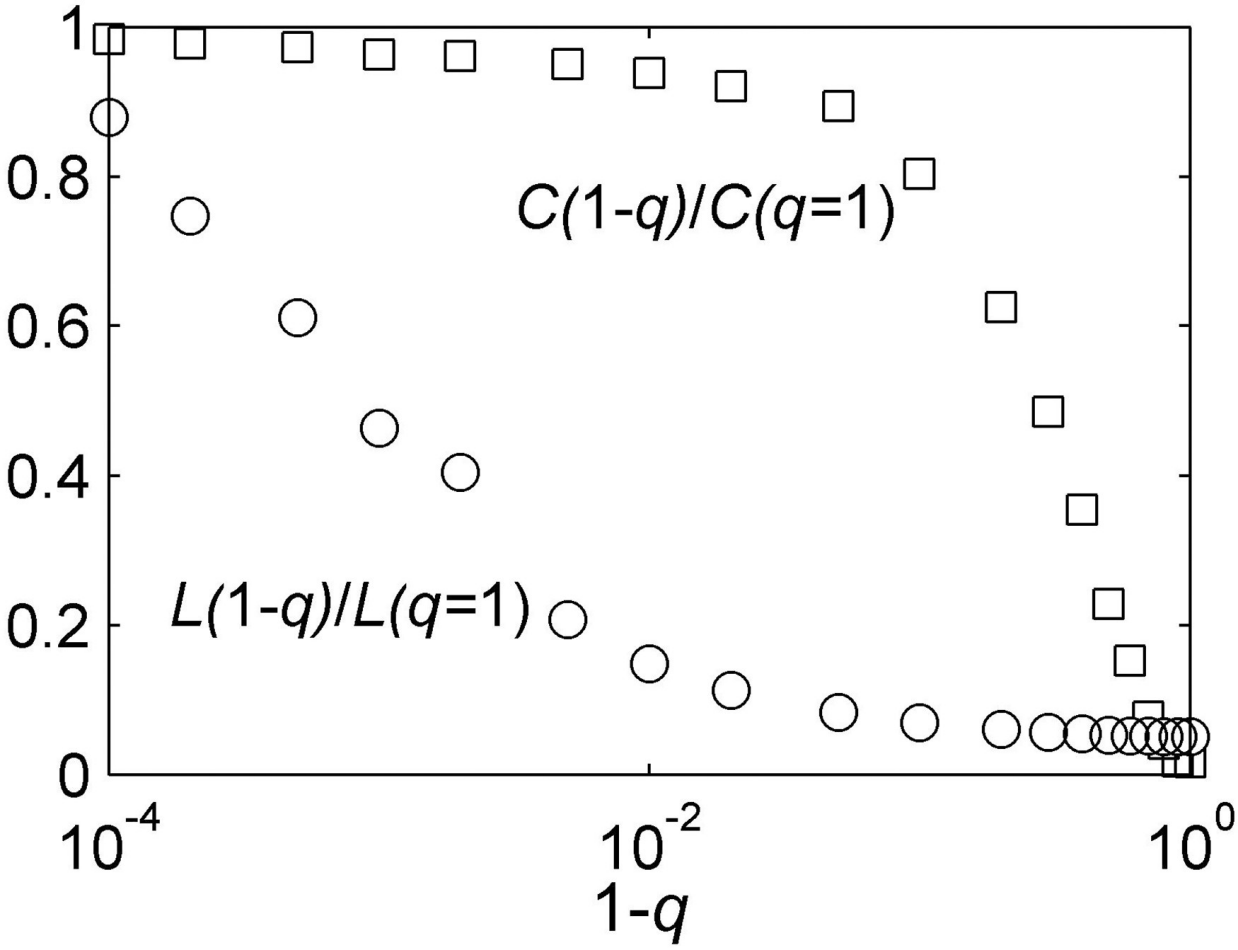
Small world characteristics. The figure shows the average clustering coefficient (*C*) and average path length (L) for networks with different homophily (*q*). The small world quotient *Q* is defined as the ratio of *C*(1-*q*)/*C*(*q* = 1) to *L*(1-*q*)/*L*(*q* = 1). If the quotient Q is greater than 1, the network has the small world characteristics. However, larger the quotient *Q*, more significant are the small world characteristics of the network. This means that, as long as *q* satisfies the condition: 0.0001 < *q* < 1, the network has a small world feature.

In fact, the network model established in this paper is fully consistent with the characteristics of small world networks. For convenience, the average path length *L* and the average clustering coefficient *C* are introduced to quantitatively analyze the small world characteristics of the network. The average path length is defined as the average number of ties in the shortest path among all individual pairs. The average clustering coefficient is defined as follows. Suppose that consumer *i* has *m*_*i*_ peers. Then, peers have at most *m*_*i*_*(m*_*i*_ *−1)/2* ties among them. Let *C*_*i*_ denote the fraction of actual ties and *C* the average of *C*_*i*_ of all consumers. The small world quotient *Q* is defined as the ratio of *C*(1-*q*)/*C*(*q* = 1) to *L*(1-*q*)/*L*(*q* = 1). The network, that has been established, is consistent with the nature of Fig 2.

In this paper, the values of homophily lie within the range of 0.1–0.9 and are fully included within this range (Fig 2), thus meeting the characteristics of small world networks.

There are two things requiring attention. Firstly, following the conventions in the marketing literature [6,8] and the process of new product proliferation, both parties will observe each other’s behavioral choices to determine their own behavior, which is in line with the reality. Therefore, it is assumed that the social ties are symmetrical, which means that the tied nodes can influence each other, whereas the social ties are symmetrical. Secondly, a correlation between the consumer’s number of links and its propensity to adopt [6] is not explicitly modeled. However, higher number of links is convenient to contact more people and achieve more information, which is propitious to adopt the new product earlier [14].

### Seeding strategies

Considering the two attributes of agents (the position in the social network and the propensity to adopt the product), three seeding targets, namely the social hubs, early adopters and randomly seeding are determined. In line with the previous literature [6,31,34], the distribution of agent’s degree is taken and the top 10% of the agents are selected as hubs, who have the largest number of social connections, and are randomly seeded with different seeding size. For example, seeding to the social hub, whereas the seeding size is 6%. Specifically, according to the social hub selection criteria, 10% of the total number of agents is selected as social hubs, and then, 60% of the hubs are randomly selected from a uniform distribution as seeds. In this way, seeding size is 6%. According to the definition of early adopters [35,36,57–60], the agents, who have the top 10% highest propensity to seed according to the seeding size, are chosen. For the randomly seeding, the seeding consumers randomly taking no consideration of those attributes (position and propensity) are selected, due to which, they represent the average customers. On the basis of seeding strategy, a new set of seeding targets is selected for each simulation run. At the start of every simulation run, agents from the three sets (social hubs, early adopters, or random seeds), are selected at a certain percentage to activate the diffusion process.

### Experimental design

At the very beginning, the agents in the market are established and everyone is assigned the three attributes (*p*, *q* and *d*) according to three normal distributions. The probability to spread negative WOM (*d*) is negatively correlated with the propensity to adopt the new product (*p*), whereas *q* represents the effect of WOM on the connections. Before the product is launched in the market, seeding program is carried out according to different seeding strategies, and the propagation process is initiated. All the adopted consumers, including the “seeds”, would spread WOM. Whether or not to spread negative WOM is determined by the parameter *d*. In each simulation period, the probability of change in state of each potential consumer will be affected by positive and negative WOMs, which can be calculated according to Eqs (1)–(6). The results were compared with the random number given by the system and decided whether to change the state or not. This process is repeated until the simulation is terminated.

In order to calculate the NPV, the profit achieved in every single simulating period is collected, and then, a 10% discount rate per period is used, which is a reasonable annual rate for many markets. In the end, all the profits are summed to obtain the NPV.

$$NPV=\overset{n}{\underset{t=1}{\sum }}a_{t}*{\left(1-r\right)}^{t-1}$$ (7)

where *t* refers to the current period, *n* is the total number of periods, *a*_*t*_ is the number of consumers adopted in stage *t*, and *r* is the discount rate. For the market penetration, the number of consumers, who have adopted the new products, are counted as a percentage of the total number of agents in the market.

$$MP=\overset{n}{\underset{t=1}{\sum }}a_{t}$$ (8)

In the long run, companies intend to occupy more market share, while in the short term, achieving more direct profit is crucial. Therefore, two performance measurements are chosen, namely the net profit value (NPV) and the market penetration (MP). For easy comparison of the three seeding strategies and to control other factors’ influences, the ratio of the performance measurements of the two diffusion processes is introduced: one process, in which the company adopts seeding activities, while the other is the raw diffusion process in the same market (all parameters are the same) without the introduction of seeding program. Specifically, the NPV ratio (NPVR) and MP ratio (MPR) are computed using Eqs (9) and (10).

$$NPVR=\frac{NPV_{\text{Seeding}}}{NPV_{\text{Base}(no\;\text{seeding})}}$$ (9)

$$MPR=\frac{MP_{\text{Seeding}}}{MP_{\text{Base}(no\;\text{seeding})}}$$ (10)

Table 2 shows the simulation parameters and the relevant references. According to the last column of Table 2, all the parameters and the ranges are selected from previously published empirical and theoretical studies. Seeding size has a decisive effect on the seeding performance. In general, previous studies used seeding sizes, which do not exceeded 10% (within the range of 2–9%) [4,32]. The subsequent work suggests that the optimal seed size is supposed to be lower [61]. Therefore, five values are chosen between 2–10% with the increments of 2%, covering all the values that would be taken in the real world. The mean values of *p* and *q*, which represent the external and internal influentials, respectively, are classical parameters and their ranges are strictly the same in this paper, which is in accordance with the previous studies [6,13,24,35,62,63]. The range of mean value of the probability of consumers spreading negative WOM is consistent with the original paper studying the effect of negative WOM in the diffusion [24]. For the level of homophily, values are set all over the theoretical range of 0.1–0.9 with the increments of 0.2 [64]. Due to the absence of a study focusing on the standard deviation of the probability of consumers spreading negative WOM, the standard deviation is set as a multiple (0.5, 1, 1.5, and 2) of the mean of the probability of consumers spreading negative WOM [8], which can cover most of the situations.

**Table 2:** Parameters and their corresponding ranges.

Parameter	No. of levels	Parameter value or range	References
Size of network	1	3000	[24]
Seeding size	5	0.02, 0.04, 0.06, 0.08, 0.1	[4,32,53];
Mean value of *p*, external influence	5	0.001, 0.006, 0.011, 0.016, 0.021	[6,13,24,35,62,63];
Mean value of *q*, internal influence	5	0.01, 0.03, 0.05, 0.07, 0.09	
Mean value of *d*, probability of consumers spreading negative WOM	5	0.05, 0.1, 0.15, 0.20, 0.25	[24];
Standard deviation of *d*	4	0.5, 1, 1.5, 2 multiple the mean value of d	[8,64];
Degree of homophily *h*	5	0.1, 0.3, 0.5, 0.7, 0.9	[64];
Simulation termination condition	1	95% of the market has made the decision, or 30 periods are finished	[6,24,35,65]
Number of simulation replication	1	100	

## Results

In the current ABMS model, eight variables are involved. They are performance with 2 measures, seeding targets with 3 options, internal influence with 5 levels, mean of the propensity to adopt with 5 values, mean of the probability of consumers spreading negative WOM with 5 values, standard deviation of the probability of consumers spreading negative WOM with 4 values, 5 levels of homophily and seeding size with 5 values. In order to eliminate the stochastic effects of simulation, each simulation is run 100 times, resulting in 2*3*5*5*5*4*5*5*100 = 7,500,000 simulation experiments. Furthermore, the simulation results are averaged over 100 replications for every parameter combination.

This section presents the analysis of results, which consists of two parts. The first part shows the optimal seeding targets on average and under different situations. In the second part, the impacts of the three conditions, the level of homophily, and the mean and standard deviation of the probability of spreading negative WOM on the performances of the three seeding targets would be studied, respectively.

In this paper, two measurements of performance are examined. They are the net present value (NPV) and the market penetration. The results analysis will use NPV performance as an example. When the corresponding results relevant to MP are different, a special explanation will be given. However, if there is no explanation, the results are similar.

### Optimal seeding targets

**Fig 3:**
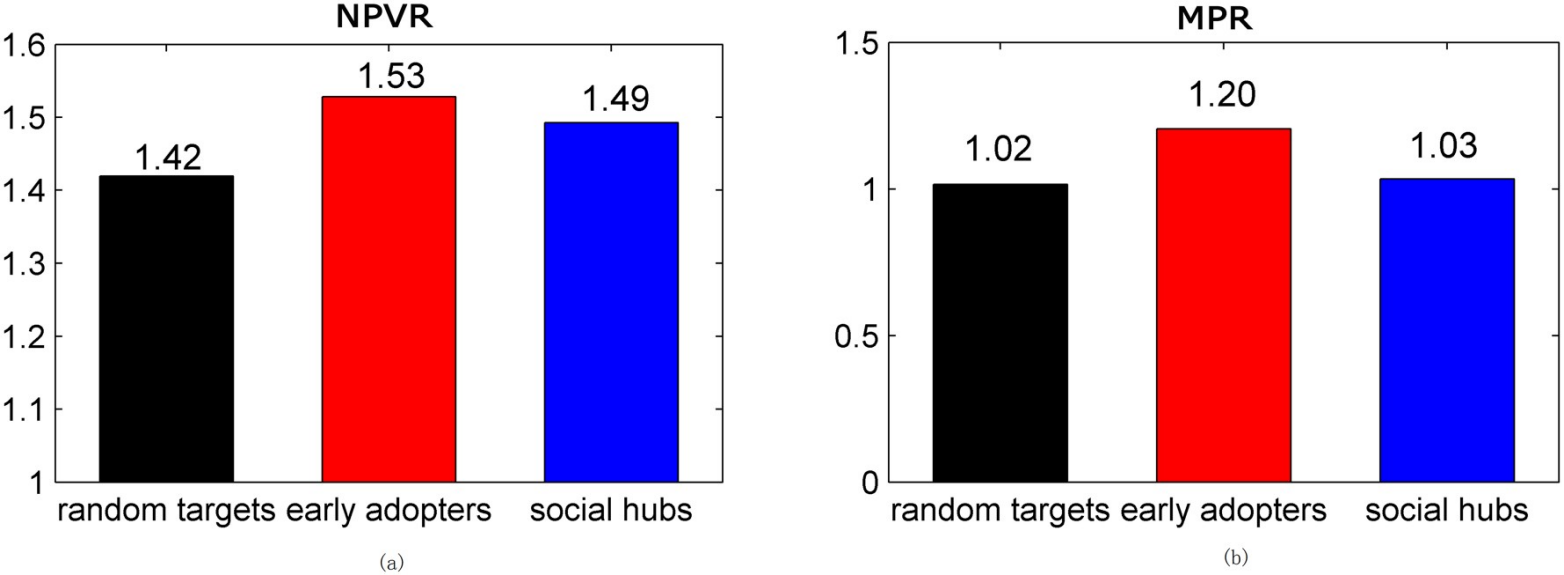
Performances of the three seeding targets. The black bar indicates the performance obtained by randomly seeding, while the red bar indicates the performance generated by seeding early adopters. The blue bar indicates the performance of seeding hubs. Figs (a) and (b) show the net present value (NPV) and the market penetration of seeding, respectively.

Firstly, the average values of NPVR for the three seeding targets are compared in the presence of negative WOM. In this work, one-way analysis of variance (ANOVA) is used to test whether there is a significant difference between the mean values of the performances generated by seeding and non-seeding. According to the results of the STATA analysis (F (3,50000) = 1416.31, p < 0.001), the *p* value is less than the significance level of 0.001, indicating that the seeding has a significant positive effect on NPV. Using the Tukey’s method in the subsequent post hoc analysis, it can be concluded that seeding early adopters can generate the highest NPVR (M = 1.528), followed by seeding hubs (M = 1.493) and randomly seeding (M = 1.419). For the part of market penetration, seeding programs can significantly enhance the MP value and seeding early adopters increase the MP value by 20.4 percent, which is obviously higher by 1.5% and 3.4% for the randomly seeding and the seeding hubs, respectively. The results are shown in Fig 3.

In order to have more realistic results, the changes in optimal seeding target are explored with changes in the three factors, the level of homophily, the mean and the standard deviation of the probability of spreading negative WOM. Some differences exist among the results related to NPV and market penetration (MP).

**Fig 4:**
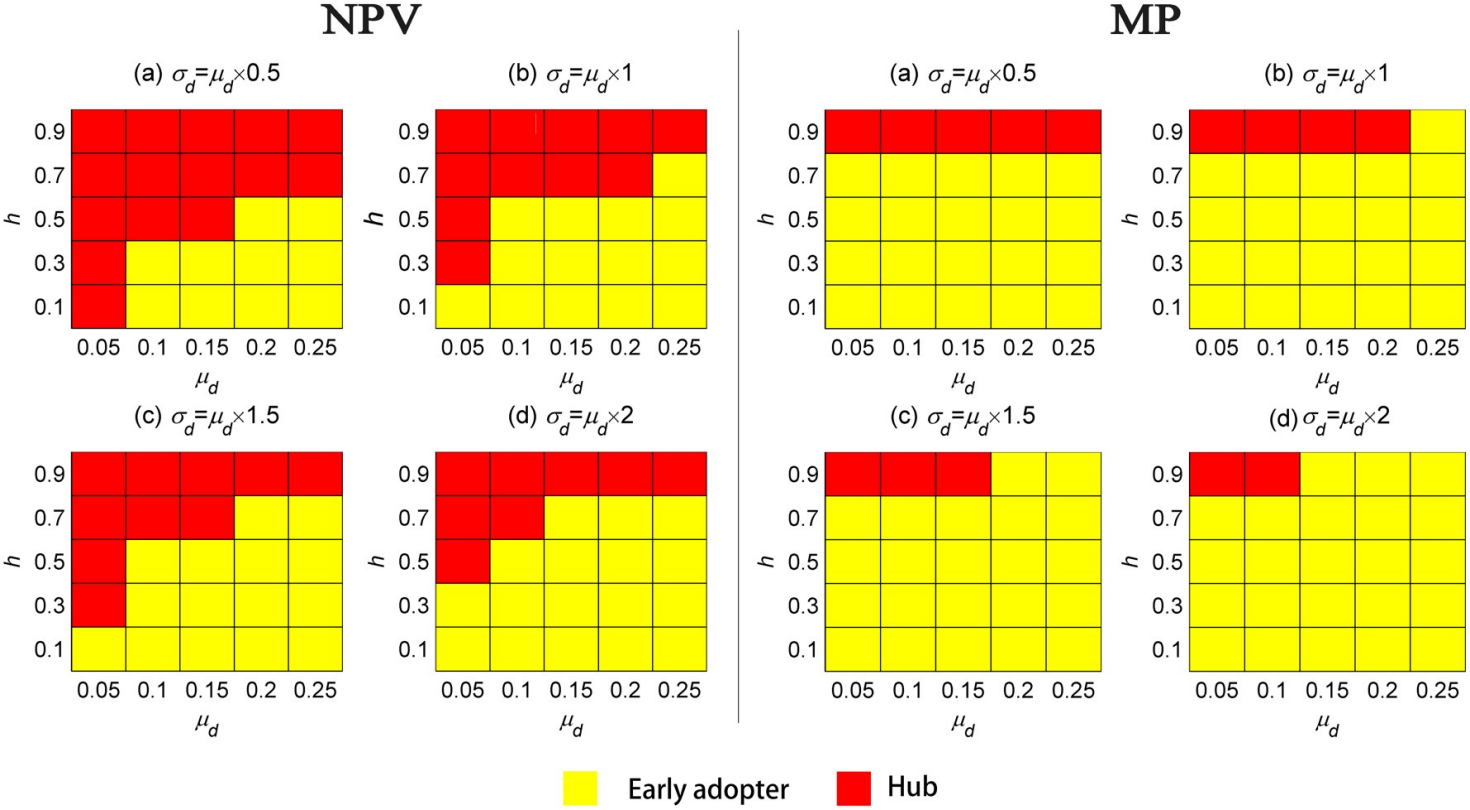
Optimal seeding targets at the combinations of three factors. Each of the above figures is determined based on the standard deviation of the probability of spreading negative WOM. The *x*-axis of each plot is the mean of the probability of spreading negative WOM, while the *y*-axis is the level of homophily. Each square corresponds to a situation of combination of the three factors. The yellow color block indicates that the optimal seeding targets under this situation are early adopters, while the red color block represents the hubs.

Early adopters are especially effective when the goal of seeding is to increase the market penetration. When the target is to optimize short-term profit, the advantage of early adopters will be weakened, because the hubs’ connection is larger, and the effect is obvious at the early stage of diffusion. The results are shown in Fig 4.

It can be seen from Fig 4 that several situations deserve our attention. When the companies pay more attention to NPV, and as the level of homophily increases, the probability that hubs will become the optimal seeding target increases, whereas the early adopters show an opposite trend. In a high level of homophily market, agents connect with others, who have similar propensity to adopt. In such a market, most of the early adopters connect with each other and have less connections with the other type of consumers, which makes it impossible for the early adopters to spread positive WOM to others, who are not early adopters [61]. When some of the early adopters are chosen as seeds, many connections may appear among them and they can only pass on the positive WOM within a finite portion of the social market [35,66,67]. In comparison, social hubs are randomly located in the network and have a large amount of connections, which can help information regarding the products propagate.

As the proportion of consumers spreading negative WOM increases, that is, the quality of products declines, the probability that seeding early adopters is the optimal strategy becomes higher than that of hubs. When the quality of product is high, seeding hubs can generate the highest NPV under more situations. There are few negative public WOM in the diffusion process. This is similar to the situation where there is only positive WOM in the market. As the quality of product declines, seeding early adopters can obtain the highest NPV with a higher probability.

When managers pay more attention to market penetration, the situation becomes much simpler. At the extreme level of homophily, hubs and early adopters may be the optimal seeding targets according to the mean and standard deviation of the negative WOM. Except for this situation, early adopters are always the optimal seeding targets. For the market penetration, negative WOM is harmful in two aspects. First, it reduces the probability of adoption of potential consumers, which slows down the propagation process. Second, rejecters, one of the sources of negative WOM, are the obstacles for the propagation and directly reduce the amount of adoption. Therefore, the original amount of negative WOM has an important influence on market penetration.

**Result 1**. On average, seeding programs can significantly improve the net present value (NPV) and market penetration (MP). In the presence of negative WOM, seeding early adopters can generate the highest NPV and market penetration, followed by seeding hubs and randomly seeding. Seeding early adopters outperforms NPV for a low-quality product and a niche product, and significantly promotes market penetration. Hubs can help increase the NPV within a high level of homophily market and for a mainstream product.

### Influence of negative WOM and homophily on seeding performance

In this section, the effect of each parameter on the NPVRs of the three seeding targets is analyzed. In this regard, three separate regressions are conducted for the three seeding targets with different dependent variables. The NPVRs of the three seeding targets are generated for the same independent variables, the mean and the standard deviation of the probability of dissatisfying targets and the degree of homophily, whereas the control variables are the mean of *p*, the mean of *q* and the seeding size. According to the previous researches [8], the impact of degree of homophily on NPVR is nonlinear. In this work, the squared term of the degree of homophily is added into the list of independent variables. Because, both the variables of the degree of homophily and the squared term appear in the regression equation, the two variables are centered to eliminate the multicollinearity. In order to test whether there is collinearity between the independent variables or not, the variance inflation factor (VIF) is calculated. Larger the VIF, more serious is the collinearity. The empirical judgment method shows that when 0<VIF<10, there is no multicollinearity, whereas for 10≤VIF<100, there is a strong multicollinearity. When VIF≥100, there is severe multicollinearity. In this regression, for all variables, the VIF is unity. The coefficients of the independent variables of three regressions are presented in Table 3.

**Table 3:** Standardized coefficients of the independent variables for three seeding targets.

*Independent variable*	*Seeding early adopters*	*Seeding hubs*	*Randomly seeding*
The mean of probability of consumers spreading negative WOM	0.084	-0.055	-0.050
The standard deviation of probability of consumers spreading negative WOM	-0.157	-0.219	-0.211
The level of homophily	-0.275	-0.036	0.012
The square term of the level of homophily	-0.032	0.018	0.025
Adjusted R-square	0.567	0.567	0.500

Notes: Dependent variable is NPVR. All reported coefficients are standardized and significant at the 0.01 level. Two variables, namely the degree of homophily and the squared term of that, are centered.

The results given in Table 3 show that: when the mean of probability of consumers spreading negative WOM increases, the relative impact (NPVR) of seeding early adopters increases and that of seeding hubs and randomly seeding both decreases. The standard deviation of WOM has a negative relationship with NPVR of the three seeding targets. Higher level of homophily will lead to lower NPVR of seeding early adopters and hubs. The relationship between the level of homophily and NPVR of randomly seeding shows a U-shape curve.

#### Impact of the average dissatisfied proportion

**Fig 5:**
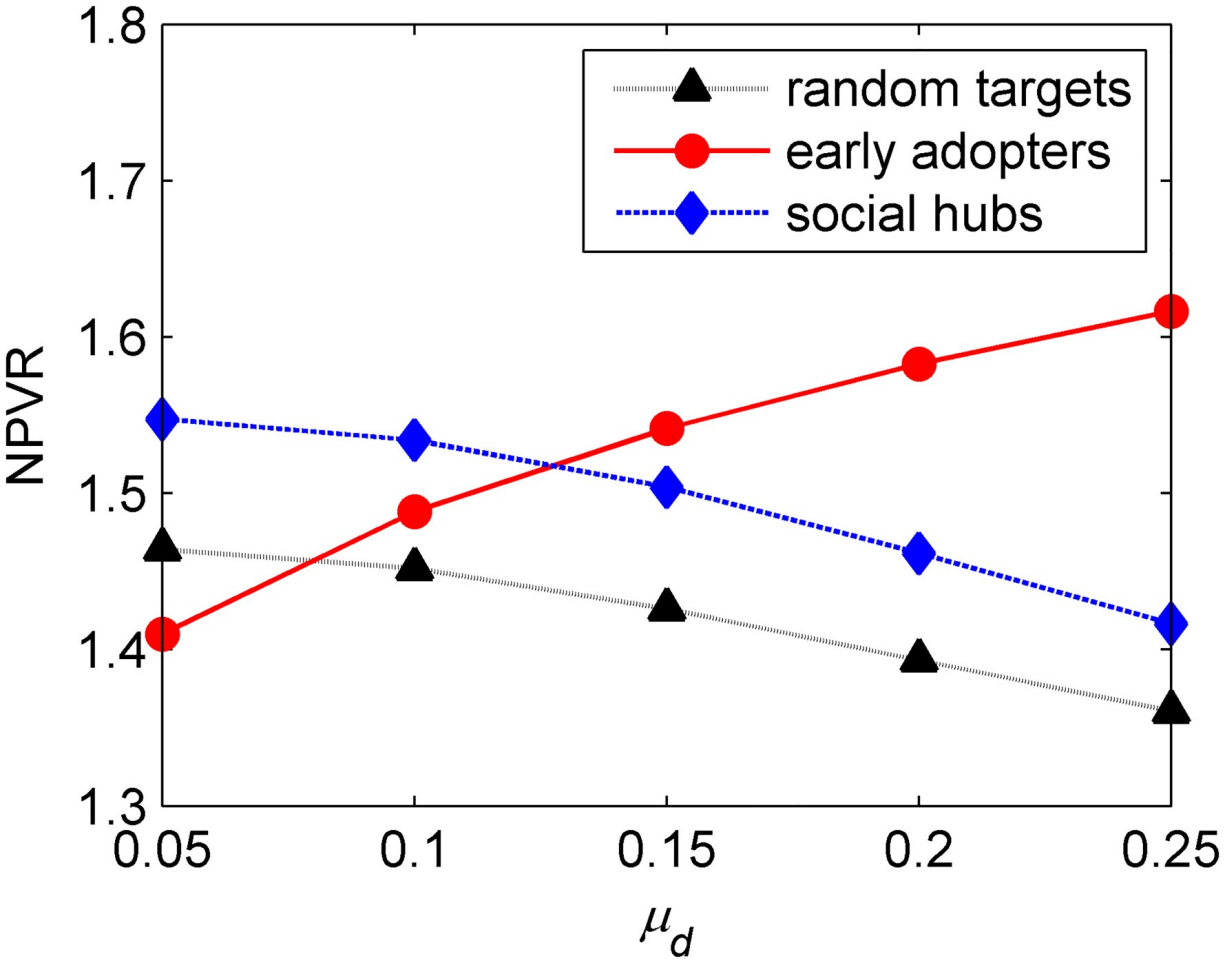
Influence of the mean value of the probability of spreading negative WOM on the three seeding targets. In Fig.5, the abscissa represents the average proportion of dissatisfaction, while the ordinate indicates the change in the ratio of net present value.

The results given in Table 3 show that, as the average dissatisfied proportion increases, which means that the quality of the product decreases, the NPVR values of both the seeding hubs and randomly seeding decrease, and this can be explained easily. However, on the contrary, the NPVR of seeding early adopters increases, as shown in Fig 5.

In fact, when the mean value increases, the number of negative WOM increases in the market, and the NPV generated by seeding or non-seeding are significantly reduced. However, the NPVR of seeding early adopters increases, no matter how much the average dissatisfied proportion increases. Since, seeding early adopters cannot only enhance the diffusion process, but can also initiate relatively smaller part of dissatisfied adopters at the same average dissatisfied proportion, as compared to no seeding, which can reduce the amount of negative WOM and improve the probability of adoption, especially for the low-quality product. For the hubs and the randomly chosen consumers, when the quality reduces, the amount of negative WOM increases and the time of existence of a certain amount of negative WOM goes up.

**Result 2**. Seeding early adopters is more effective for improving the NPV and MP for a low-quality product, wherein adopters are more likely to spread negative WOM.

#### Impact of the standard deviation of probability of dissatisfied adopters

**Fig 6:**
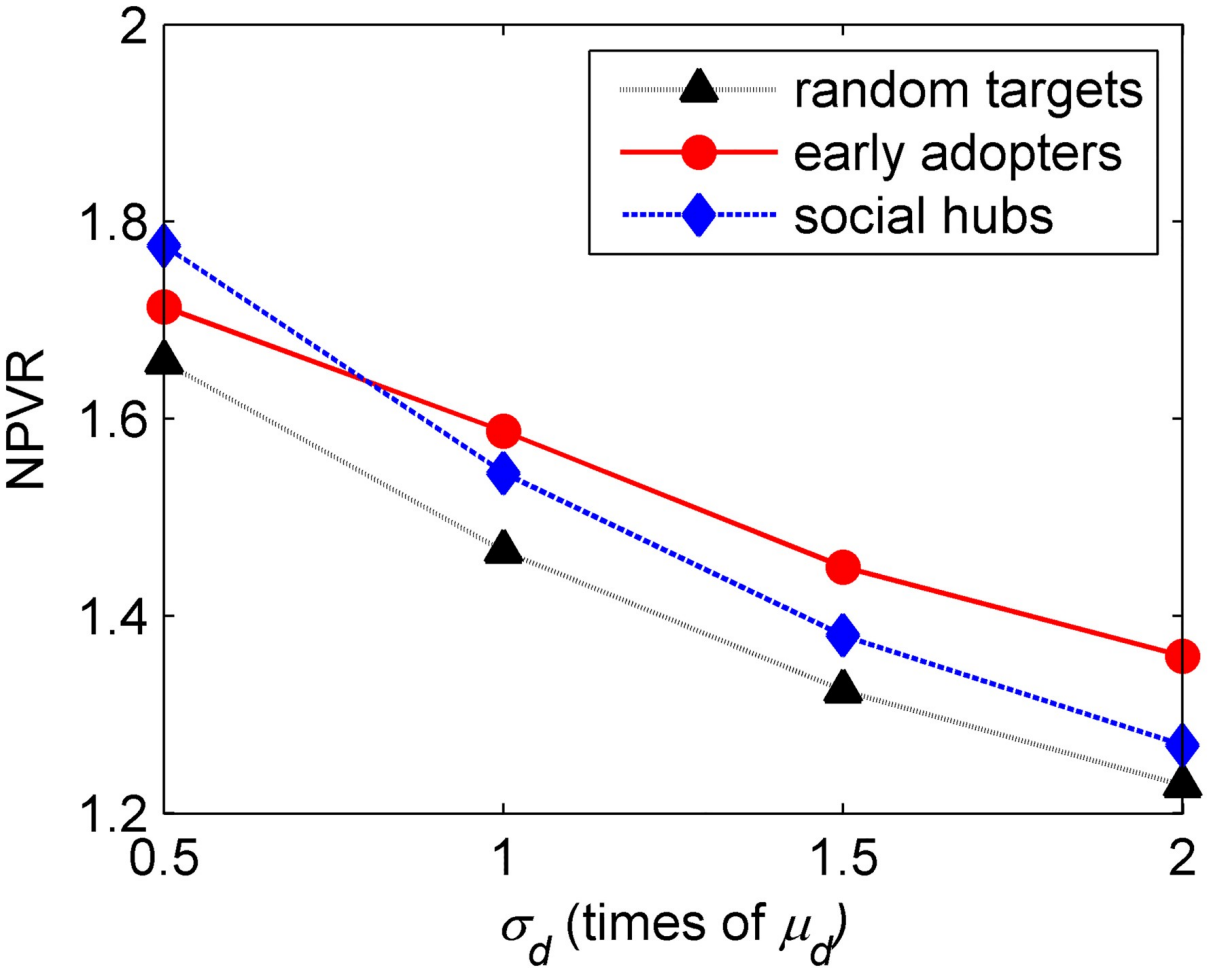
Impact of standard deviation of probability of consumers spreading negative WOM on the three seeding targets. In Fig.6, the abscissa represents the standard deviation of the probability of dissatisfaction, while the ordinate represents the change in the ratio of net present value.

The results given in Table 3 show that the standard deviation of probability of dissatisfying negatively impacts the NPVR for the three seeding targets. According to Fig 6, as the standard deviation increases, it means that the characteristic of the new product changes from mainstream to niche, the NPVR of seeding early adopters decreases, though the rate of decrease is slower than that of the other seeding targets.

A low standard deviation denotes small differences among probabilities, which means that the consumers are dissatisfied about the product. When the standard deviation increases, the differences increase. The mean of the probability of consumers spreading negative WOM of the early adopters is low. On the contrary, the mean of the probability of consumers spreading negative WOM of the late adopters, who have low propensity to adopt, is high. On account of high propensity, the early adopters are hardly to be triggered as rejecters, especially in the early periods. For the late adopters, when the standard deviation increases, more negative WOM is contagious and more potential consumers have a higher probability to become rejecters. According to the constant mean of dissatisfied proportion, the proportion of negative WOM in the whole market is same regardless the standard deviation. However, higher standard deviation leads to more rejecters in the earlier periods. Therefore, the NPVR generated by each seeding target decreases with the standard deviation of the dissatisfied proportion.

However, the mean of the probability of dissatisfying of the part of early adopters, who have low probability of being dissatisfied, decreases. Therefore, seeding early adopters can decrease the initial dissatisfied proportion when the standard deviation of the probability of dissatisfying increases. That is why, the NPVR of seeding early adopters decreases slowly.

**Result 3**. When the degree of specialization of the new product increases (niche product), the performance of seeding decreases. The NPV generated by seeding early adopters decreases slower.

#### Impact of homophily

**Fig 7:**
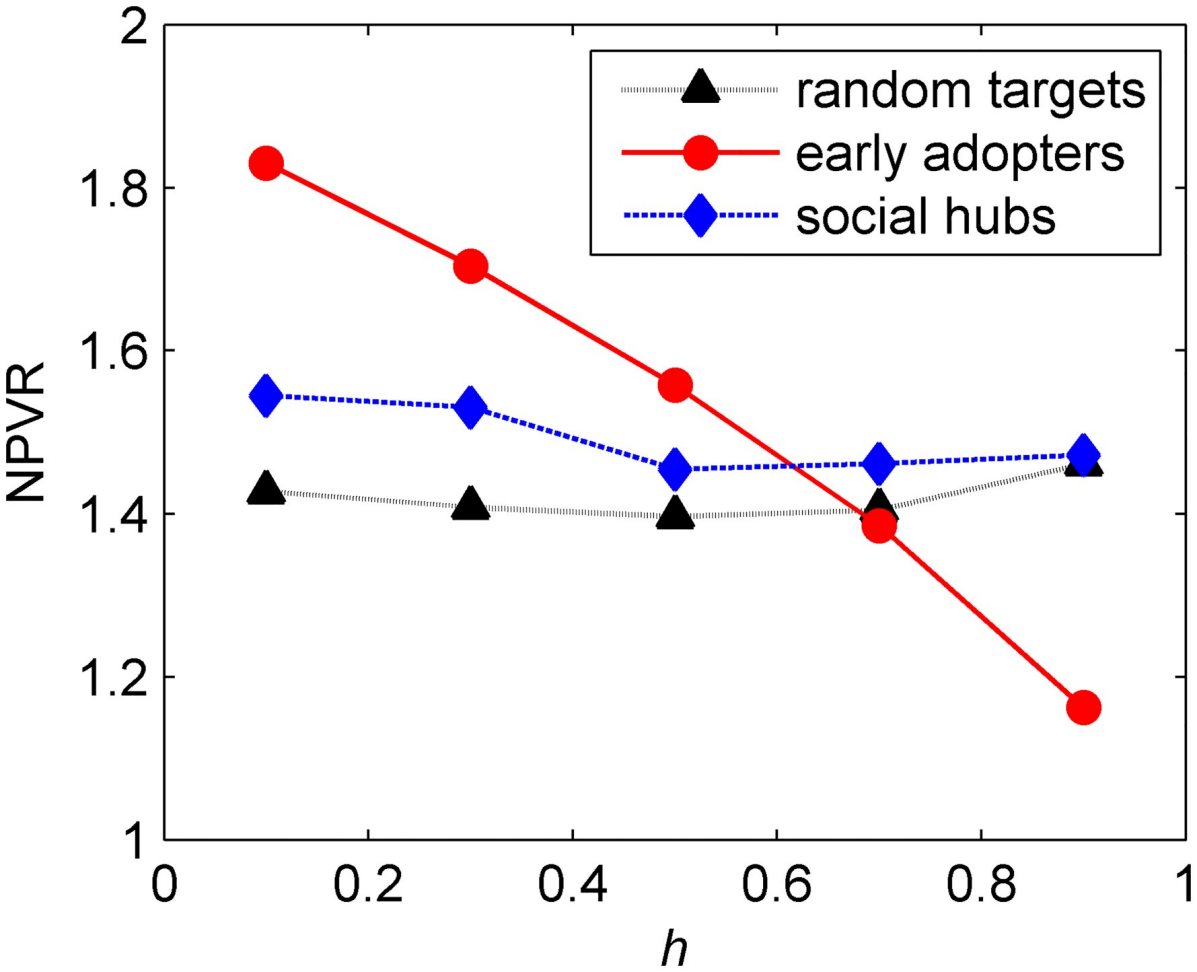
Impact of levels of homophily on the NPVR for three seeding targets. In Fig.7, the abscissa represents the level of homophily, while the ordinate represents the change in the ratio of net present value.

In this section, the influence of homophily is explored. According to the results shown in Fig 7, as the degree of homophily increases, the NPVR of seeding early adopters decreases and that of seeding hubs and random targets show a U-shape relationship.

Firstly, the relationship between the NPV generated by seeding early adopters and the level of homophily is affirmatory. The degree of homophily impacts the distribution of early adopters, and then, impacts the propagation capacity of WOM from early adopters. When the level of homophily increases, the early adopters form clusters and the positive WOM can transmit only within a finite zone of the market. As a result, as the degree of homophily increases, the impact of seeding early adopters on the NPV decreases.

Social hubs are randomly located in the network regardless of the degree of homophily. A certain proportion of hubs with low propensity to adopt are chosen regardless of the degree of homophily. As the degree of homophily increases, the number of agents with low propensity connected with this proportion of hubs will increase, which generates a considerable amount of negative WOM in the early period within the area of the late adopters. Along with the low propensity, these late adopters are easier to become rejecters, who have a significant negative influence on the diffusion process. However, the negative effects gradually reduce. This is due to the reason that, when the degree of homophily increases, seeding the social hubs is more effective than non-seeding. When the degree of homophily is extremely high (0.9), the NPVR of seeding hubs is a little higher than that when the degree of homophily is 0.7. Although the U-shape is not significant on average, it can be found that seeding hubs can help diffusion process when the degree of homophily is extremely high (0.9), as compared to other seeding targets. The results are somewhat different from those reported in previous research, and it can be found from the analysis that the reason is the negative WOM in the network.

For the randomly seeding, when the level of homophily is from low to high, more rejecters will appear at an earlier stage, which is consistent with the results for seeding hubs. However, the relationship is somewhat different for randomly seeding when the level of homophily is from a moderate to high. This is due to the reason that the randomly selected targets have substantially less connections compared to hubs. Therefore, considerably less numbers of adopters with low propensity are initialed in the early periods. At the same time, randomly choosing can also help product transfer among the clusters resulting from high homophily. In summary, the level of homophily and the NPVR of randomly seeding show a U-shape relationship, which is in line with the previously reported results [8]. Therefore, the effect of negative WOM on NPVR is higher for seeding hubs compared with the seeding hubs.

**Result 4**. Higher level of homophily will lead to lower NPVR generated by seeding early adopters and hubs. The relationship between the level of homophily and NPVR of randomly seeding shows a U-shape.

## Discussion and implications

With the presence of negative WOM, large-scale agent-based simulations are conducted to seek the optimal seeding targets on average and in a reasonable market scenario, which is determined by a set of values of three selected factors. From both the short-term and long-term perspectives, the results confirm that seeding programs can significantly enhance the net present values (NPV) and the market penetration. According to the position of consumers in the social network (social hubs) and the propensity to adopt (early adopters), proper seeding targets are chosen and their performances are compared with those of no seeding and randomly seeding. Due to the lack of studies focusing on the impact of negative WOM on the seeding performance, the results present novel insights into choosing the optimal seeding target in the presence of negative WOM.

### Implications of the research

First, when the negative WOM is taken into consideration, seeding programs still can significantly enhance the NPV in the short run and market penetration in the long run for most simulated cases. On average, seeding early adopters generates the highest NPV and market penetration, followed by seeding hubs and randomly seeding. Early adopters, with higher propensity to adopt, rate the product more favorably and have a higher probability to spread positive WOM. Hubs, having the most social ties with others, can speed up the diffusion process. Of course, whether seeding is done or not, and regardless of the seeding target, the rules for the contagion of new products are first spread from early adopters, followed by hubs, and then, to everyone. Seeding early adopters accelerates the initial phase of the spread of new products, while spreading a higher proportion of positive WOM to social networks. Seeding the hubs allows the product to spread to consumers throughout the network in a short period of time, thus generating a large number of negative WOM and resulting in the appearance of rejecters, who are a hindrance to the spread of new products and have heavier influence than the positive WOM. Therefore, seeding early adopters is the optimal strategy in the presence of negative WOM.

Seeding them can help increase the amount of positive WOM and decreases the number of rejecters in the early periods, which is beneficial to the diffusion process. However, hubs with low propensity to adopt have a high probability to spread negative WOM. Due to the large number of connections, the negative WOM from hubs would influence massive potential consumers.

Three factors, namely the mean of probability of spreading negative WOM, the standard deviation of probability of consumers spreading negative WOM, and the level of consumer homophily have a great impact on which type of consumers are the most promising seeding targets. In addition, different goals of firms, net present value and market penetration also impact the selection of the most appropriate seeding target.

The mean of probability of consumers spreading negative WOM indicates the quality of the product. When it is low, small amount of negative WOM will be spread in the social network, which approximates to the case without the presence of any negative WOM, which has already been studied in previous works. In this case, seeding hubs generate the highest NPV. However, for the market penetration, early adopters are still the optimal seeding targets. When the mean of probability of consumers spreading negative WOM increases, seeding early adopters can enhance NPV and market penetration. However, the NPV and market penetration generated by seeding hubs and randomly seeding would decrease with the increase in mean value.

Higher standard deviation of probability of consumers spreading negative WOM shows that the consumers with high propensity to adopt (early adopters) have the higher mean value of probability of consumers spreading negative WOM and the consumers with low propensity to adopt (late adopters) have the lower mean value of probability of consumers spreading negative WOM. In other words, some consumers love this product, while the others hate it, in which case, it is called a niche product. When the standard deviation increases, the relative impact of seeding programs decreases. When the standard deviation is low, seeding social hubs is the best choice to improve the NPV, whereas the early adopters are the optimal targets when the standard deviation is high for both the NPV and the market penetration. Actually, seeding early adopters generates the most market penetration, no matter what the standard deviation is.

The relationship between the level of homophily and the performance of seeding hubs is different with the presence of negative WOM compared to the situation without the presence of negative WOM. Without negative WOM, seeding hubs generate higher NPV with the increase in the level of homophily from moderate to high level. When the negative WOM in the social network is taken into consideration, the NPV generated by seeding hubs decreases. Due to the presence of plenty of connections, negative WOM from the social hubs can influence a huge proportion of potential consumers. Because of the high level of consumer homophily, most of the peers connected with the hubs, who spread negative WOM, have low propensity to adopt, and thus, have higher probability to become rejecters, who are always viewed as the “obstacles” in the product’s diffusion process.

### Managerial implications

Most of the new products launched fail every year and only about 3% achieve a great success. Negative WOM is believed to be one of the most important reasons. Seeding program is a popular and effective method to speed up the diffusion process of a new product. Taking the negative WOM into account, this paper provided a more comprehensive and more realistic study about choosing the optimal seeding target from the short-term and long-term perspectives. The results firstly lend strong support for the conclusion that seeding program can still enhance the NPV and market penetration with the presence of negative WOM and provides guidelines for optimal seeding target under various scenarios of market, which include firm’s business objectives, quality of the product, target customers for the product, and consumers’ homophily.

From the short-term perspective, firms pursue high net values as the business objective. When the quality of new product is high, seeding hubs is the best choice to enhance the NPV, which is in line with the findings reported in previous studies. If the new product is mainstream, which is designed to pander to a wide range of consumers’ tastes, seeding hubs can also generate the highest NPV compared to seeding early adopters and randomly seeding. However, when the quality is not good enough, early adopters become the most promising targets to seed. Seeding early adopters can delay the emergence of negative WOM and decrease the amount of negative WOM in the early periods. Similarly, when the new product is designed for a unique type of customers, called a “niche product”, seeding early adopters gains the highest NPV. The level of consumer homophily has an important impact on the selection of seeding target. When the level of homophily is low, consumers connect randomly, and the NPV obtained by seeding early adopters is the highest. As the level of homophily increases, consumers tend to connect with people similar to themselves. As a result, the seeded early adopters have a great possibility to connect with each other with fewer connections to other types of consumers. Therefore, they cannot spread positive WOM to others. In this case, seeding hubs generates the highest NPV, though it is not much better than the NPV gained by randomly seeding. At the same time, if the cost of recognizing hubs is high, randomly seeding can replace seeding hubs.

From the long-term perspective, market penetration is the ultimate goal of firms. With the presence of negative WOM, firms should take actions to delay the emergence of negative WOM and decrease the quantity and coverage area of negative WOM, which can enhance the probability of adoptions and decrease the number of rejecters. Based on the former analysis, seeding early adopters can help achieve this goal. In addition, according to the results, seeding early adopters has overwhelming superiority compared to seeding hubs and randomly seeding, except for one case when the level of homophily is extremely high. In this case, early adopters connect with each other and have fewer connections with others, so that the positive WOM cannot propagate to a large area. The difference of the market penetration generated by seeding hubs and randomly seeding is little, which are both lower than that of seeding early adopters.

Firms are sometimes not clear about the situations, such as the level of homophily, the quality expected by the consumers and the tastes of the consumers. In these cases, according to the results, it is suggested that the NPV and market penetration generated by seeding hubs are always not the lowest one compared to other seeding targets and no seeding programs.

## Limitations and future research

In this work, the negative WOM is taken into account, which extends the research on seeding. There still exist several limitations in this study, and some future research directions are proposed accordingly. In fact, the sources of WOM are varied, such as the social ties, observational learning, and social norms. The WOM is acquainted from social ties, which is in line with the majority of related studies. Future study can capture more channels of WOM. The performances of the three separated seeding targets are compared, and in each seeding program, only one of the targets is selected. In future, studies can compare the performances of mixed multiple targets. Some adopters may not spread WOM to others, especially for the dissatisfied adopters. Alternatively, these adopters spread WOM to only a few peers or the timing of spreading WOM is delayed. Future work can do some more complicated empirical research about this issue. When simulating the diffusion process, it is assumed that the consumers cannot change the states after they have made a decision. Actually, the rejecters may change their mind (state) due to the increased positive WOM received in the later periods. Changing this assumption may help find more interesting insights. Three factors that have impact on the diffusion process are taken into consideration. There may be some other factors, which can influence the contagious process. Future researches can consider the influencing factors more comprehensively.

## Supporting information

S1 Data DataS1.zip. Raw data and simulation programs to obtain the raw data. Processed data, which can be used directly for analysis.
(RAR)

S2 Data Data description. **README.docx**. A detailed description of each file in **DataS1.zip**.
(PDF)
