# Supplementary material for: Seeding strategies for new product launch: The role of negative word-of-mouth
Source: PLoS One. 2018 Nov 5;13(11):e0206736. doi: 10.1371/journal.pone.0206736 (PMC6218060; doi:10.1371/journal.pone.0206736)
Supplement: S2 Data — README.docx. A detailed description of each file in DataS1.zip. (PDF) [file pone.0206736.s002.pdf]

Gallo, T., M. Fidino, E.W. Lehrer, S. Magle. Mammal diversity and metacommunity dynamics in urban green spaces: implications for urban wildlife conservation. ***Ecological Applications***

Data S1

R script, JAGS model, and data used to estimate γ-, α-, and β-diversity and community and population dynamics in urban green spaces in the Chicago metropolitan area, IL USA.

Author(s)

Travis Gallo
Lincoln Park Zoo, Urban Wildlife Institute, Department of Conservation and Science
2001 N. Clark St., Chicago, IL 60614
tgallo@lpzoo.org

Mason Fidino
Lincoln Park Zoo, Urban Wildlife Institute, Department of Conservation and Science
2001 N. Clark St., Chicago, IL 60614
mfidino@lpzoo.org

Elizabeth W. Lehrer
Lincoln Park Zoo, Urban Wildlife Institute, Department of Conservation and Science
2001 N. Clark St., Chicago, IL 60614
llehrer@lpzoo.org

Seth Magle
Lincoln Park Zoo, Urban Wildlife Institute, Department of Conservation and Science
2001 N. Clark St., Chicago, IL 60614
smagle@lpzoo.org

File list (files found within DataS1.zip)

```
gallo_et_al_2017_site_type_analysis.R
gallo_et_al_2017_site_type_model.R
gallo_et_al_2017_y_array.RDS
gallo_et_al_2017_z_array.RDS
gallo_et_al_2017_j_matrix.RDS
```

`gallo_et_al_2017_site_type.txt`
`gallo_et_al_2017_species_names.txt`
`gallo_et_al_2017_urban_cov.txt`

File descriptions

`gallo_et_al_2017_site_type_analysis.R` – the only file that you need to open. Loads datasets and formats data for JAGS model. Has code for summarizing the posterior distributions and calculating derived alpha, beta, and gamma diversity.

`gallo_et_al_2017_site_type_model.R` - JAGS model used to estimate metacommunity and metapopulation dynamics and derive diversity metrics. Read in `gallo_et_al_2017_site_type_analysis.R`.

`gallo_et_al_2017_y_array.RDS` - The number of days each species was detected and is supplied as data to the JAGS model so that each species detection probability can be calculated. This array is ordered by species by site by season. Species are in the same order as `gallo_et_al_2017_species_names.txt` and sites are in the same order as `gallo_et_al_2017_j_matrix.RDS`.

`gallo_et_al_2017_z_array.RDS` - Data on whether or not each species were observed at the 100 camera trapping sites each season. If they were detected the cell takes a value of 1, if they were not detected it takes a 0, and if the site was not sampled it takes an NA. This is a species by site by season array in the same orders as `gallo_et_al_2017_y_array.RDS`.

`gallo_et_al_2017_j_matrix.RDS` - Data on the number of days a camera trap was active at each site and season. Used with the detection data to calculate detection probabilities, and is a site by season matrix. If a site was not sampled a zero is reported.

`gallo_et_al_2017_site_type.txt` - A design matrix indicating the land use category of each site.

`gallo_et_al_2017_species_names.txt` - A vector of the observed species used in our metacommunity analysis.

`gallo_et_al_2017_urban_cov.txt` - The urbanization covariate for each site, in the same order as all of the other data.

Note: All of the files in DataS1 must be within your working directory for the analysis to work. Our analysis was done in parallel and used all but two of the cores in a computer. Therefore, if

you have two or less cores on your computer you will need to adjust the MCMC settings annotated within `gallo_et_al_2017_site_type_analysis.R`.
